# Genetic and Environmental (Co)variation of Egg Size, Fecundity, and Growth Traits in Arctic Charr

**DOI:** 10.1111/eva.70135

**Published:** 2025-07-18

**Authors:** Paul Vincent Debes, Sabine Brigitte Céline Lobligeois, Einar Svavarsson

**Affiliations:** ^1^ Department of Aquaculture and Fish Biology Hólar University Sauðárkrókur Iceland

**Keywords:** egg size, fecundity, genetic correlation, life‐history trait, reproductive success, trade off

## Abstract

Egg size and fecundity are both positively associated with maternal reproductive success, yet maternal resource limitations result in a trade‐off between these two traits. Exploring this trade‐off, the extent of genetic and environmental influences on egg size and fecundity and of correlations between these and other traits, and thus, the effects acting within vs. among generations is therefore a central goal in both evolutionary ecology and selective breeding. Using multi‐generational captive Arctic charr ( *Salvelinus alpinus* ) records, we quantified genetic and environmental effects on and correlations between egg size and fecundity, body size (a proxy for growth) and condition prior to maturation, and body size at maturation. We estimated that genetic contributions to variation in egg size and fecundity are moderate to high. Egg size and fecundity do not significantly correlate at the genetic level but do correlate negatively at the environmental level. Growth prior to maturation and size at maturation are positively correlated with fecundity and egg size at the phenotypic level. Genetic correlations with growth are positive for both egg size and fecundity but weaker for egg size. Contrarily, the environmental correlations with growth are of the opposite sign, also weaker for egg size, and increasing growth leads to decreasing egg size but increasing fecundity. Consequently, reproductive success can be optimized across generations via independent selection responses of egg size or fecundity and by correlated selection responses with body size. Ultimately, the egg size‐fecundity resource trade‐off in Arctic charr is resolved via growth‐controlled phenotypic plasticity acting within generations.

## 1 Introduction

Offspring size and fecundity are important reproductive traits in both nature and captivity because they often affect offspring quality and number, respectively, and thereby reproductive success (Anderson and Gillooly 2021). In egg‐laying animals, larger offspring usually hatch from larger eggs, and this maternal effect on offspring quality often has fitness consequences for both offspring and parents. A larger offspring size leads to a higher parental reproductive success because, everything else being equal, larger offspring usually have a higher fitness (fish: Einum et al. 2004; insects: C. W. Fox and Czesak 2000; birds: Krist 2011). Furthermore, a higher fecundity leads to a higher parental reproductive success because, everything else being equal, more eggs usually result in more offspring surviving. However, the offspring size and number trade off by virtue of maternal resource constraints (Lack 1947), such as energy and egg‐holding space (reviewed by Zera and Harshman 2001), and the optimization of this trade‐off depends on the environment (D. A. Roff 2002; Rollinson and Hutchings 2013b; Smith and Fretwell 1974; Stearns 1992). In harsh offspring environments (e.g., low resource availability, high predation, and high competition), theory and data suggest that parents maximize reproductive success by reducing fecundity and increasing egg size, whereas in benign environments, it is maximized by producing many small offspring (Einum and Fleming 1999; Rollinson and Hutchings 2013a, 2013b). Surprisingly, for most life‐history traits, the nature of trade‐offs is not fully understood, particularly whether they arise from genetic or environmental factors (Chang et al. 2024; D. A. Roff 1996). Importantly, a trade‐off at the genetic level can limit the selection response of traits across generations, whereas a trade‐off at the environmental level can limit trait expression within generations.

A complexity in understanding the trade‐off between egg size and fecundity endures because there is little clarity on the extent of direct vs. indirect genetic or environmental effects on variation of egg size or fecundity via additional traits covarying with egg size, fecundity, or both (de Jong and van Noordwijk 1992; Jonsson and Jonsson 1999; Pincheira‐Donoso and Hunt 2017; Schroderus et al. 2012). Understanding multivariate patterns of variance and covariance is especially important because a negative correlation between two fitness‐related traits that share a common resource is often used as evidence for a trade‐off (A. A. Agrawal et al. 2010), although the correlation between two traits can be affected by additional, covarying traits (Pease and Bull 1988; Van Noordwijk and De Jong 1986). Additional traits covarying with egg size, fecundity, or both may be maternal traits that are assumed to act as maternal predictors for offspring environmental quality or reflect variation in the absolute amount of maternal resources available to produce eggs (reviewed by Mousseau and Fox 1998). For example, the maternal growth rate experienced in a mother's early environment has been suggested to be a predictor of the environmental quality that her offspring will experience (Burton et al. 2020; Einum and Fleming 1999; Jonsson et al. 1996; Scott 1962; Taborsky 2006). An expectation is that females who grew more rapidly (in benign environments) produce smaller and more eggs because the optimal allocation strategy in benign environments is producing smaller and more offspring (Burton et al. 2020; Quinn et al. 2004; Reznick et al. 1996; Thorpe et al. 1984). As another example, maternal body size at a specific age may covary with maternal resources available at that specific age, and which may affect both egg size and fecundity (de Jong and van Noordwijk 1992). An expectation is that larger females with more resources will produce both larger and more eggs (de Jong and van Noordwijk 1992; Kamler 2005). When also considering female size variation caused by differences in age rather than by differences in the growth rate at the same age, size variation will, to a large extent, covary with developmental duration, that is, with age at maturation (e.g., Roff 2000; D. A. Roff 2002). Here, an expectation is also that older (and larger) females produce larger and more eggs than younger (and smaller) females (Kamler 2005). However, it is not always clear whether variation in body size that may covary with egg parameters is the result of variation in growth rate until the same age or of different developmental durations, although the growth rate and maturation age are different traits. Finally, for females of the same body size (length), or for females that are statistically standardized to the same size, variation in body condition may reflect variation in *relative* maternal resource amount available for reproduction (C. T. Marshall et al. 1999; but see Koops et al. 2004). Thus, understanding trade‐offs between two traits requires considering additional traits, some of which may be expressed at different developmental stages (Blows 2007; Walsh and Blows 2009).

In fishes, understanding whether and how genetic and environmental effects influence traits related to reproductive success is not only interesting from evolutionary and ecological perspectives but also critical for applied fields such as conservation biology, fisheries, and aquaculture. For example, in conservation biology, relationships between the maternal body size and egg size or fecundity have heavily been debated due to uncertain knowledge about inadvertent evolutionary vs. environmentally plastic effects on changes in means and (co)variances of these traits (Araki et al. 2008; T. D. Beacham 2003; Terry D. Beacham 2011; Christie et al. 2012; Fleming et al. 2003; Fleming and Gross 1990; Charles W. Fox and Heath 2003; Heath, Heath, et al. 2003; Heath, Moya‐Laraño, and Fox 2003; Matos 2012; Quinn et al. 2004). Likewise, in fisheries research, maternal age, size, and body condition are known modifiers of the relationship between current spawning stock biomass and future fish stock size, that is, reproductive success (Kell et al. 2015; Koops et al. 2004; D. J. Marshall et al. 2021; van Deurs et al. 2023). Knowing whether effects are genetic or environmental has thus implications for sustainably managing fish stocks, also to avoid fisheries‐induced evolution that compromises population viability (Heino et al. 2015; Hutchings and Fraser 2008). In aquaculture, the egg size may or may not be of production efficiency interests to selective breeding because egg size may or may not covary with early offspring survival in hatcheries (Brooks et al. 1997; Jónsson and Svavarsson 2000). These conflicting results may not be reflected by perceptions of aquaculture producers, where often a strong belief prevails that larger eggs enhance offspring survival and thus recruitment (own experience; Brooks et al. 1997). In contrast, because fecundity is usually high in fishes to support aquaculture production it has been suggested to be surveyed for indirect selection via other traits (T. Gjedrem and Baranski 2009; Gjerde 1986) or to be a relevant trait when selecting adequate founder populations (Bromage et al. 1990). Central questions relevant to many fields of research are thus first how a maternal resource trade‐off is approached from evolutionary and ecological perspectives, involving both genetic and environmental effects, and second how strong genetics and environmental effects act on egg size and fecundity directly and indirectly via additional traits.

In the current study, we quantify first the genetic and environmental contributions to (co)variance in egg size and fecundity, and second how the two traits expressed at sexual maturity covary with prior growth conditions (approximated by body length and mass) and condition at age 2 years, and additionally with the body size at sexual maturity (body mass after egg stripping). The age of 2 years is in this case 1 or 2 years prior to sexual maturity. We thereby test whether a trade‐off between egg size and number arises at the genetic or environmental (plasticity) level and how the trade‐off is affected by growth traits at different ages. To do so, we use multi‐generational data of a pedigreed Arctic charr ( *Salvelinus alpinus* ) population from the Icelandic breeding program that we analyze with a multivariate animal model.

## 2 Materials and Methods

### 2.1 Study Population

Data records used are for cohorts 1990–2021 of the Icelandic Arctic charr breeding program population kept at the breeding nucleus in Hólar í Hjaltadal, northern Iceland. The Arctic charr is an iteroparous, anadromous fish of the family Salmonidae that is known for its high growth potential at low water temperature and its high phenotypic plasticity; a mature female spawns once during an annual period (Klemetsen et al. 2003). The breeding program population was kept indoors at the local daylight cycle in a flow‐through freshwater system mixing cold and warm degassed and oxygenated water from local borehole and mountain‐runoff sources. The water temperature varied seasonally 2°C–10.5°C. Fish were automatically fed *ad libitum* using commercial diets. It is an admixed population, and nine Icelandic lakes and rivers have successfully contributed founders between 1990 to 2003. Each of the 32 cohorts analyzed consisted initially of an average of 190 families. The first 30 cohorts followed a mating design resulting in predominantly paternal half‐sib families. The most recent included cohort (2021) followed optimum contribution criteria for mating and also involved maternal half‐sib families. At first feeding, each family (standardized to ≤ 400 individuals) was maintained in one blue 15 L tank until large enough for individual tagging (> 5 g). From mating until tagging, family number was reduced to an average of 135 families based on lowest body deformity and highest survival rates and average body mass. Early familial survival rate in the hatchery may relate to how well the experimenter‐decided egg stripping date matched the female ovulation date (Gillet 1991); we thus do not expect this pre‐selection to have systematically affected any studied maternal trait, but we can also not fully exclude this possibility via inadvertent selection for other non‐considered traits that may be correlated with the studied maternal traits. Individual tagging at the age of several months proceeded initially by using Floy tags (cohorts 1990–1992), a combination of branding and fin clipping (cohorts 1993–2015), and using passive integrated transponder tags (since cohort 2016). After a full‐sib‐tank‐period of several months and tagging, an average of 29.5 fish from each family were combined into two large tanks (31 and 36 m^3^) and commonly grown until age 2 years. Additional fish of each family were grown and assessed at external aquaculture station(s) but their records do not contribute to the current study. At age ~2 years, the maturation status was assessed based on external characteristics, and fork length, wet mass, and a binary score for body deformity presence were recorded. Fish were subsequently either culled or pre‐selected as broodstock candidates (600–1000 individuals per cohort). Pre‐selection until cohort 2019 was based on a breeding value index for body mass and maturation probability at age 2 years. Maturation probability was assessed based on maturation binaries of fish at the nucleus and test stations or sex‐specific maturation scores of lethally assessed siblings at the test stations that used to get converted to binaries until cohort 2019 and have been used directly thereafter. Since cohort 2019, the pre‐selection index additionally includes breeding values for body length and condition at age 2 years and for age‐at‐maturity of pre‐selected individuals (3, 4, or 5 years). Furthermore, only fish maturing at age ≥ 4 years have been allowed to reproduce since cohort 2019. The spawning period between 2004 and 2019 (the cohorts with reproduction trait records) lasted on average 43 days and fell between 26^th^ September and 8^th^ November. Choosing mating partners among selected broodstock has proceeded with the limitation to keep inbreeding of resulting offspring < 5% and proceeds since cohort 2017 to minimizing inbreeding. The most current four cohorts that contributed data (2017–2021) have been selected for an average of 8.9 generations (based on pedigree information), have an inbreeding coefficient of *F*_*i*_ = 0.024 (calculated based on the diagonal of the pedigree‐based additive relationship matrix minus one, generated as reported below), and an effective population size of *N*_*e*_ = 165 (calculated based on individual inbreeding change across generations following Gutiérrez et al. 2009).

### 2.2 Trait Records

After we removed data records from fish that expressed body deformities, got mature at age 2 years, or that showed up as statistical outliers that indicated data errors (Appendix A in Supporting Information), we utilised up to 122,642 individual (male and female) records from a total of 4320 families of the cohorts 1990 to 2021 for body mass (±1 g) and length (±1 mm) at age 2 years. We calculated Fulton's body condition following Heincke (1908) as $K=\frac{\text{mass}\;\left(g\right)}{\text{length}\;{\left(\mathrm{cm}\right)}^{3}}\times 100$. For up to 2815 females of the cohorts 2004 to 2019 from a total of 706 families and being exclusively first‐time spawners (egg traits may differ between first‐time and repeat spawners; Kamler 2005) at age 3 or 4 years, we also measured total egg wet mass (including ovarian fluid; measurement precision: ±1 g) and eyed egg wet mass (±1 mg, average of 25 eggs per family, averaged across family means if a female contributed to several families). For the same females and cohorts, we also measured stripped body mass (±1 g) but missed 2017 cohort records. We predicted average “green” (unfertilized) egg mass via regression coefficients of average green egg wet mass on average eyed egg wet mass that we measured for 31 females of the 2019 cohort (Appendix B). We then estimated fecundity (number of eggs) by dividing the total egg mass by average green egg mass.

We regarded variation of the (log of) body length of immature individuals at age 2 years as a proxy of variation for the somatic growth rate and variation of body condition at age 2 years as a proxy of composite variation for body reserves and body shape prior to initiating maturation. Specifically, at a common age, length variation may reflect the sum of variation in past somatic growth, whereas mass variation may serve as a compound for variation for past somatic growth, amount of energy stores, and body shape, whereby the latter two are commonly captured by the body condition index used here.

We log‐transformed (ln) all traits except body condition to stabilize variances and analyze proportional rather than absolute differences (absolute differences vary temporally and spatially). Furthermore, the traits relate to each other and to body condition linearly on the log scale, so that the transformation resulted in more meaningful correlation estimates. We additionally scaled (dividing by the standard deviation) and mean‐centered all traits to facilitate model convergence but reported results as either proportional differences or on the original scale via the respective back transformations.

### 2.3 Fitted Model

We fitted one multivariate animal model to the six response traits of egg mass, fecundity, stripped body mass, body length, condition, and mass. Using one common multivariate model is advantageous compared to several univariate models because it allows estimating (co)variances among traits and may reduce selection bias in parameter estimates. Specifically, records for female reproductive traits at ages 3 and 4 years were influenced by selection bias. Within cohorts, a bias existed because reproductive trait records only existed for females selected first for breeding values of body mass at age 2 years, which correlated with the female reproductive traits (see results), and second for breeding values for maturation probability across sexes expressed at age 2 years. Across cohorts, a bias may have existed because records for reproductive traits have been recorded only since cohort 2004, whereas the recording of other traits and selective breeding started already for cohort 1990. To reduce these biases on model estimates, we fitted multivariate animal models including all breeding station records for body mass contributing data to the selection decisions (Henderson 1975; Meyer 1991; Mrode 2013; Pollak et al. 1984).

In the model, we included several fixed effect terms; some were of direct interest, and all effects resulted in more general estimates for the variance components. Specifically, we fitted fixed factorial cohort effects to all traits to remove variation due to systematic differences in environmental quality and hatchery practices among years but common to each cohort. Please note that trends due to genetic change across cohorts can be predicted separately from such systematic environmental effects in an animal model. Furthermore, we fitted fixed factorial maturation age effects (3 or 4 years) to the three response traits expressed at maturity that we interacted with the cohort effects to capture the additional systematic environmental differences among years experienced by the different maturation ages of each cohort (4‐year‐old females would have experienced one additional year of environmental effects compared to 3‐year‐old females). Thus, we here treated maturation age as a nuisance trait and thereby “removed” its variation along with all the covariation of traits expressed at maturity. Specifically, in fishes, egg mass and fecundity are usually positively associated with female age or with the increase in female size that comes with age (reviewed by Kamler 2005). However, the relationship between egg mass and fecundity may change with age if one trait increases stronger than the other trait, which we also directly assessed. Importantly, when not accounting for age effects, one may obtain results that are driven by the different rates of trait change with age, which may not represent resource trade‐offs within individuals (see Discussion).

Furthermore, when differences in the relationship between egg mass and fecundity exist among founder populations, a common analysis without population effects may lead to estimating correlations between traits that reflect contributions from differences among populations rather than the correlations between traits within individuals. It is indeed well known that unaccounted genetic differences among founder populations can lead to biased estimates of (co)variance parameters of an admixed population (Westell et al. 1988; Wolak and Reid 2017). Thus, because our data were influenced by temporally varying genetic contributions of several founder populations, we fitted the animal model with (the inverse of the) additive relationship matrix augmented for genetic group effects (Westell et al. 1988) that we calculated using the “ainverse” function of the R‐package ASReml‐R v. 4.2.0.332 (Butler et al. 2018) and which is effectively similar to fitting fixed population‐contribution continuous covariates (reviewed by Wolak and Reid 2017).

We fitted random terms for (i) full‐sib effects (*f*), (ii) common environmental effects (*c*), (iii) additive genetic effects (*a*), and (iv) residual effects (*r*). Full‐sib effects combined possible family‐specific environmental (tank) effects acting during the first few months after hatching until tagging, maternal effects, and dominance effects. Common environmental effects combined the environmental effects common to individuals within a mixed‐family holding tank that followed the family‐specific rearing tank period until data recording at age 2 years and the time‐of‐record‐taking effects. Additive genetic effects reflected the expected sum of additive genetic loci on a trait and were estimated via a link to the inverse of the additive relationship matrix based on the pedigree (*A* ^−1^). Residual effects combined the random environmental individual effects and measurement error. Please note that the genetic effects predicted under an augmented relationship matrix, *A* ^−1^*, correspond to the genetic value due to both within and among population genetic effects. The (co)variance of the random effects among traits was modelled according to either

$$\Sigma_{1}=\left[\begin{array}{cccccc} \sigma_{\mathrm{egg}\text{size}}^{2} & \sigma_{\mathrm{egg}\text{size},\text{fecundity}} & \sigma_{\mathrm{egg}\text{size},\text{strip}.\text{mass}} & \sigma_{\mathrm{egg}\text{size},\text{length}} & \sigma_{\mathrm{egg}\text{size},\text{condition}} & \sigma_{\mathrm{egg}\text{size},\text{mass}}\\ \sigma_{\text{fecundity},\mathrm{egg}\text{size}} & \sigma_{\text{fecundity}}^{2} & \sigma_{\text{fecundity},\text{strip}.\text{mass}} & \sigma_{\text{fecundity},\text{length}} & \sigma_{\text{fecundity},\text{condition}} & \sigma_{\text{fecundity},\text{mass}}\\ \sigma_{\text{strip}.\text{mass},\mathrm{egg}\text{size}} & \sigma_{\text{strip}.\text{mass},\text{fecundity}} & \sigma_{\text{strip}.\text{mass}}^{2} & \sigma_{\text{strip}.\text{mass},\text{length}} & \sigma_{\text{strip}.\text{mass},\text{condition}} & \sigma_{\text{strip}.\text{mass},\text{mass}}\\ \sigma_{\text{length},\mathrm{egg}\text{size}} & \sigma_{\text{length},\text{fecundity}} & \sigma_{\text{length},\text{strip}.\text{mass}} & \sigma_{\text{length}}^{2} & \sigma_{\text{length},\text{condition}} & \sigma_{\text{length},\text{mass}}\\ \sigma_{\text{condition},\mathrm{egg}\text{size}} & \sigma_{\text{condition},\text{fecundity}} & \sigma_{\text{condition},\text{strip}.\text{mass}} & \sigma_{\text{condition},\text{length}} & \sigma_{\text{condition}}^{2} & \sigma_{\text{condition},\text{mass}}\\ \sigma_{\text{mass},\mathrm{egg}\text{size}} & \sigma_{\text{mass},\text{fecundity}} & \sigma_{\text{mass},\text{strip}.\text{mass}} & \sigma_{\text{mass},\text{length}} & \sigma_{\text{mass},\text{condition}} & \sigma_{\text{mass}}^{2} \end{array}\right]$$

or

$$\Sigma_{2}=\left[\begin{array}{cccccc} \sigma_{\mathrm{egg}\text{size}}^{2} & \sigma_{\mathrm{egg}\text{size},\text{fecundity}} & \sigma_{\mathrm{egg}\text{size},\text{strip}.\text{mass}} & 0 & 0 & 0\\ \sigma_{\text{fecundity},\mathrm{egg}\text{size}} & \sigma_{\text{fecundity}}^{2} & \sigma_{\text{fecundity},\text{strip}.\text{mass}} & 0 & 0 & 0\\ \sigma_{\text{strip}.\text{mass},\mathrm{egg}\text{size}} & \sigma_{\text{strip}.\text{mass},\text{fecundity}} & \sigma_{\text{strip}.\text{mass}}^{2} & 0 & 0 & 0\\ 0 & 0 & 0 & \sigma_{\text{length}}^{2} & \sigma_{\text{length},\text{condition}} & \sigma_{\text{length},\text{mass}}\\ 0 & 0 & 0 & \sigma_{\text{condition},\text{length}} & \sigma_{\text{condition}}^{2} & \sigma_{\text{condition},\text{mass}}\\ 0 & 0 & 0 & \sigma_{\text{mass},\text{length}} & \sigma_{\text{mass},\text{condition}} & \sigma_{\text{mass}}^{2} \end{array}\right]$$

with *f* ~ MVN (0, $\Sigma_{1}⊗F$), *c* ∼ MVN (0, $\Sigma_{2}⊗C$), *a* ∼ MVN (0, $\Sigma_{1}⊗A^{-1*}$), and *e* ∼ MVN (0, $\Sigma_{1}⊗R$), where *F*, *C*, and *R* are the respective identity matrices and *A* ^−1^* is the inverse of the pedigree‐derived augmented additive relationship matrix since the founder generation that includes 373,217 entries (plus genetic groups).

The covariance between traits expressed at age 2 years and traits expressed at age 3 or 4 years for *c* in $\Sigma_{2}$ was set to zero for several reasons. Specifically, (i) fitting a model with $\Sigma_{1}⊗C$ did not converge; (ii) the interpretation of *c* differs between age 2 years and ages 3 or 4 years; and (iii) the variance attributed to variation among *c* was relatively small (2%–6% of total phenotypic variance; see results). To elaborate on point (ii), environmental tank effects up to age 2 years may influence traits at all focal ages, but the effects related to time‐of‐record‐taking are limited to age 2 years. The reason for these time‐of‐record‐taking effects is that, for cohorts 2004–2019, the timing of record taking at age 2 years differed between replicate tanks by as much as 78 days (see Results).

The model for the response (*y*) for each reproductive trait expressed at age 3 or 4 years was:

(1) y~trait+Cohort+Age+Cohort:Age+f+c+a+e

and for each trait expressed at age 2 years:

(2) y~trait+Cohort+f+c+a+e

where *trait* is a model constant (trait specific intercept, which is adsorbed into the genetic group effects) and other terms are described as above. Please note that one common model was fitted to the six response traits, but that effects in (1) or (2) can be specified separately for each response trait in the one common model. The model was fitted in R v. 4.3.2 (R Core Team 2023) using REML under the average information algorithm as implemented in the ASReml‐R “asreml” function.

### 2.4 Model Result Processing

Based on the fitted model, we extracted several parameters and visualized the results using the R “plot” function. To allow the model‐based prediction of mean effects (and their differences) with or without genetic trend due to selective breeding (the latter corresponded to the base population genetic value plus environmental cohort effects) and, if applicable, age effects, we re‐fitted the model with genetic group effects as fixed covariates representing the genetic contribution of populations per individual and using *A* ^−1^ instead of *A* ^−1^*. The covariate approach is equivalent to fitting *A* ^−1^* but facilitates predictions of fixed effect combinations from the model. We predicted the overall averages of founder population contributions across cohorts. For each of the three reproductive traits, we predicted differences between maturation ages while averaging across those cohorts with data for both maturation ages (data for female maturation age of 3 years are limited to cohorts 2005 to 2016).

We calculated the proportions of total phenotypic variance per trait by dividing each trait‐specific variance component by the sum of all trait‐specific variance components. We calculated correlations between two traits as the ratio of the between‐trait covariance divided by the square root of the product of the two trait‐specific variances. We calculated predictive slopes of trait y based on trait x as the ratio of the between‐trait covariance divided by the variance of trait x. We obtained standard errors for all variance‐derived parameters using the delta method via the ASReml‐R “vpredict” function.

We tested for differences in average founder population genetic effects by using pairwise *t*‐tests adjusted for family‐wise multiple comparisons using the false discovery rate (*fdr*; Benjamini and Hochberg 1995). We standardized the founder population means to the environmental means expressed for the 2019 cohort (which was the latest cohort with reproductive records at the time of analysis) and for female reproductive traits to the age of 4 years (because 3‐year‐old mature females are now rare). This setting reflects how expected founder population means would be expressed under the current breeding station practices but without any genetic gain from selection since the foundation. Because we conducted this analysis based on individuals that were kept in a common‐garden environment for up to nine generations (i.e., the breeding nucleus), these average genetic founder population effect estimates should be unaffected by possible natural environmental effects varying among the ancestral population. As a limitation, most population contributions were low due to few initial breeders, and records for female reproduction traits were available after several generations of population interbreeding and selection; thus, the detection ability for founder population differences was probably relatively low. We predicted mean effects, their standard errors, and standard errors of differences among means via the ASReml‐R “predict” function. We obtained a compact letter display for multiple comparison depiction based on the “multcompLetters” function of the “multcompView” R‐package (Graves et al. 2024).

## 3 Results

### 3.1 Cohort, Maturation Age, and Cohort‐By‐Maturation Age Effects

**FIGURE 1:**
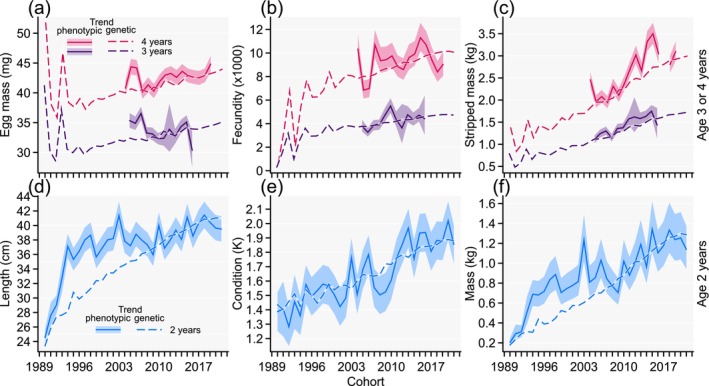
Multivariate animal model estimates for phenotypic trends (solid lines with semi‐transparent approximate 95% confidence intervals) and additive genetic trends (dashed lines) for egg mass (a), fecundity (b), and body mass after egg stripping (c) of females that are first‐time spawner at either 3 or 4 years, or for average body length (d), condition (e), and mass (f) of immature males and females at age 2 years. Depicted genetic trends have been mean standardized to the trait‐ and age‐specific environmental effects across cohorts 2007–2021 to reflect current procedures. The difference of the phenotypic from the genetic trend reflects environmental deviations. Accuracies of breeding values underlying the genetic trends are reported in Appendix C (Figure A1).

**FIGURE 2:**
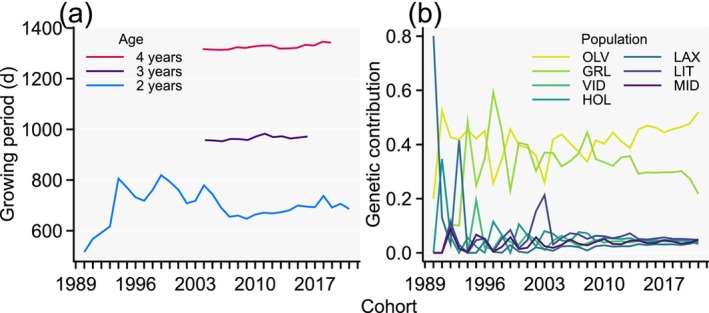
Age‐specific growing period in days (d) (a) and average genetic contributions by seven founder populations (b) across cohorts. Contributions by two additional founder populations were minor (< 1%) and are not shown.

Phenotypic trends for focal traits expressed at age 2 years (body length, condition, and mass) showed an overall increase with increasing cohorts (Figure 1), consistent with selection on larger body mass that we performed across generations (the generation interval increased from 3.2 years to 4 years). However, length, and to some extent mass, was larger in earlier cohorts than the more current cohorts (Figure 1a,f). This observation does not reflect unsuccessful selection for larger body size but can be accredited to inconsistent timing for data recording that resulted in considerably longer growing periods between first feeding and record taking in some earlier cohorts than the more current cohorts (Figure 2a). The body condition was not similarly affected by different growing periods and increased consistently across cohorts (Figure 1e). Altogether, because growing period affects both length and mass in indeterminately growing fishes, the phenotypic differences in mean values for these traits between cohorts with different growing periods may not be informative about selection responses. However, differences between maturation ages for cohort‐adjusted female traits expressed at maturity and their phenotypic means across cohorts can still be generalized because maturation time of the year (and thus growing period until trait recording) has remained relatively consistent across cohorts (Figure 2a). For focal traits expressed at maturity at age 3 or 4 years, an increasing phenotypic trend across cohorts was visually apparent for stripped body mass but less so for egg mass and fecundity. Furthermore, no trait expressed at maturity showed much cohort‐trend synchrony between maturation ages 3 and 4 years (maturation‐age‐specific solid lines in Figure 1a–c), indicating that the effects acting the year prior to maturation affected expression of these traits; that is, cohort‐by‐maturation age effects may have existed.

All tested fixed effect terms, reflecting differences in genetic‐trend‐controlled environmental effects, were statistically significant, including cohort, maturation age, and cohort‐by‐maturation‐age interactions (Table 1). At age 2 years, cohort effects were most strongly expressed for length and mass during the period 1990 to 2007, reflected in the strongest deviations of the phenotypic from the genotypic trend and that fell into the abovementioned period with considerably longer growing periods relative to more recent cohorts (Figures 1d,f, 2a).

**TABLE 1:** *F*‐test results of environmental fixed‐effect terms based on a multivariate animal model for six traits expressed in mature females at age 3 or 4 years or in immature males and females at age 2 years.

Trait	Expression age	Term	DF	DDF	*F*	*p*
Egg mass	3 or 4	Cohort	15	17.6	4.2	0.003
Fecundity	3 or 4	Cohort	15	16.5	5.7	0.001
Stripped body mass	3 or 4	Cohort	14	16.1	6.8	< 0.001
Body length	2	Cohort	31	29.4	13.5	< 0.001
Body condition	2	Cohort	31	28.1	2.4	0.012
Body mass	2	Cohort	31	28.9	7.8	< 0.001
Egg mass	3 or 4	Age	1	2737.9	2548.0	< 0.001
Fecundity	3 or 4	Age	1	2598.3	2394.0	< 0.001
Stripped body mass	3 or 4	Age	1	2519.5	4348.0	< 0.001
Egg mass	3 or 4	Cohort‐by‐Age	11	2685.7	15.7	< 0.001
Fecundity	3 or 4	Cohort‐by‐Age	11	2598.3	7.6	< 0.001
Stripped body mass	3 or 4	Cohort‐by‐Age	11	2491.9	14.3	< 0.001

Averaged across those cohorts with data records for both maturation ages (cohorts 2005 to 2016), maturation age had very strong positive and highly significant effects on egg mass, fecundity, and stripped body mass. Specifically, 4‐ relative to 3‐year‐old mature females had 19% (95% CI: 18%–20%) larger egg mass, 80% (60%–102%) higher fecundity, and 55% (44%–67%) larger stripped body mass. When standardized to stripped body mass, this corresponded to predictive slopes per 1% maturation‐age‐related body mass increase of 0.35% egg mass increase and 1.45% fecundity increase. Thus, maturation age effected an ontogenetic allometry, that is, the phenotypic relationship between egg mass or fecundity and body mass was changing with age, and thereby also the relationship between egg mass and fecundity; importantly, relative to the increase of body mass with age, egg mass experienced negative and fecundity positive allometry.

There were some strong initial fluctuations of the genetic trends (dashed lines in Figure 1) for especially the female traits that fell into the time of varying genetic contributions from founder populations (Figure 2b). However, these strongly fluctuating initial genetic trends were based on breeding values with relatively low accuracy for the female traits that lacked phenotypic records for the initial period (Appendix C; Figure A1). For later cohorts, there were overall increasing genetic trends for all focal traits (Figure 1) and these trends were based on breeding values with relatively high accuracy (Appendix C; Figure A1).

### 3.2 Genetic and Environmental Contributions to the Population Variance

**FIGURE 3:**
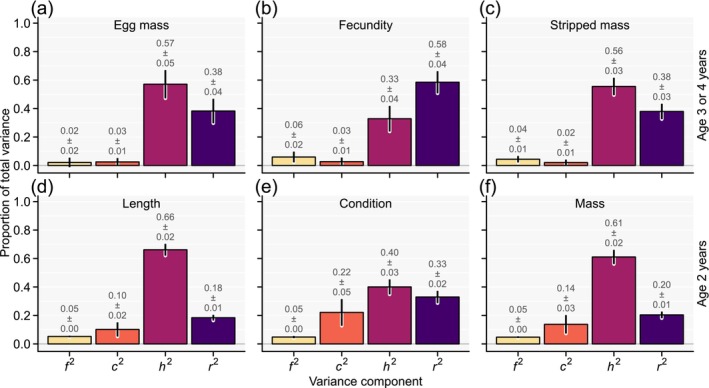
Multivariate animal model estimates for proportion of phenotypic variance, explained by effects for full sibs (*f* ^*2*^), common environments (*c* ^*2*^), additive genetic values (*h* ^*2*^), and residuals (*r* ^*2*^) for egg mass (a), fecundity (b), and body mass after egg stripping (c) of females that are first‐time spawner at either 3 or 4 years, or for average body length (d), condition (e), and mass (f) of immature males and females at age 2 years. Estimates with standard error are given as text whereas depicted error bars show approximate 95% confidence intervals.

Adjusted for fixed effects as reported above, we estimated the proportional contribution to the phenotypic variance by full‐sib (*f* ^*2*^), common environmental (*c* ^*2*^), additive genetic (*h* ^*2*^), and random environmental (*r* ^*2*^) effects for the six focal traits (Figure 3; full covariances matrices with standard errors in Appendix D; Tables A1–A5). All six traits exhibited additive genetic and random environmental variance that was clearly different from zero, and which together explained the largest proportion of phenotypic variance per trait. Full‐sib effects explained small amounts of phenotypic variance, albeit the estimates were significantly different from zero for all traits except egg mass (2%–6%; Figure 2; Table A3). Contributions by common environmental effects were, with 2%–3%, also relatively minor for the three traits expressed at sexual maturity but reached 10%, 22%, and 14% for body length, condition, and mass expressed at age 2 years, respectively. However, for traits recorded at age 2 years but not for traits recorded at sexual maturity of 3 or 4 years, common environmental effects for especially the earlier cohorts also encompassed different growing periods between tanks until data recording (in addition to different growing periods among cohorts, as reported above). Thus, contribution estimates by common environmental effects for traits recorded at age 2 years were “inflated” (and estimates of other components “deflated”) by hatchery practices related to the time of record taking between tanks during earlier cohorts. The estimated proportional contribution to the phenotypic variance by additive genetic effects, that is, heritability, was relatively high for body mass, length, stripped mass, and egg mass (0.57 to 0.66), and lower for body condition and lowest for fecundity (0.40 and 0.33, respectively; Figure 3).

### 3.3 Phenotypic, Genetic, and Environmental Correlations Between Traits

**FIGURE 4:**
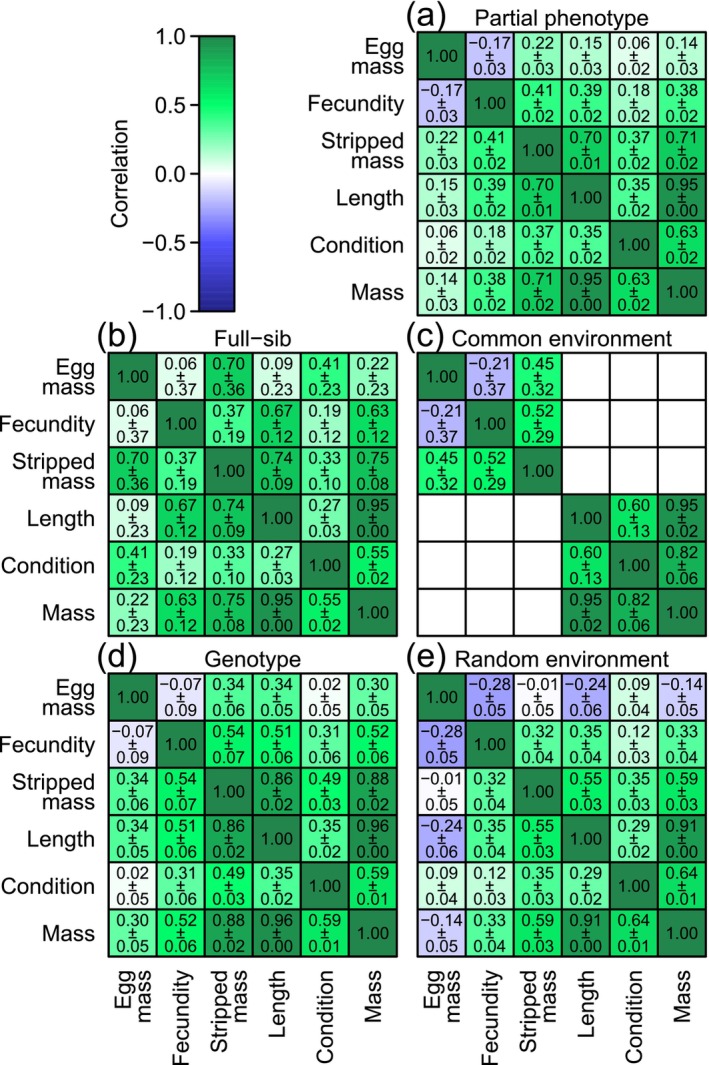
Multivariate animal model correlation estimates (± standard error) between six focal traits at the level of partial phenotype (a), full sib (b), common environment (c), genotype (d), and random environment (e).

Controlled for fixed effects as reported above, we estimated the partial correlations between traits at the phenotypic, full‐sib, common environmental, additive genetic, and random environmental levels (Figure 4). At the phenotypic level (Figure 4a), egg mass and fecundity correlated negatively, although weakly, as may be expected under a weak resource trade‐off between the two traits. This negative correlation was most apparent at the common and random environmental levels (Figure 4c,e) but close to zero at the full‐sib and additive genetic levels (Figure 4b,d). Considering the relative contribution to the phenotypic variances of the two traits by mostly genetic and random environmental effects (Figure 4a,b), the presence of a negative phenotypic correlation between egg mass and fecundity can largely be accredited to the covariance of the random environmental effects. At this random environmental level (Figure 4e), egg mass was also negatively correlated with growth prior to maturation (approximated by both body mass and length) but the correlation estimates between egg mass and stripped body mass, or body condition, were statistically not different from zero (i.e., the estimates ±2 times the standard errors included zero). In contrast, fecundity was at the random environmental level positively correlated with all growth‐related traits, including body condition. Thus, the environmental correlations with growth traits were different between egg mass and fecundity, and when considering growth prior to maturation, they had opposite signs. Contrarily, all growth traits including body condition correlated positively with each other at all considered levels. Correlation estimates at the genetic level (Figure 4d) were positive between all traits, except between egg mass and fecundity and egg mass and condition, which were statistically not different from zero. Genetic correlation estimates between egg mass and growth traits were generally lower than between fecundity and growth traits. Genetic correlations were high among traits representing growth prior to and size at maturation, and these traits genetically correlated high to moderately with body condition.

### 3.4 Founder Population Effects

**FIGURE 5:**
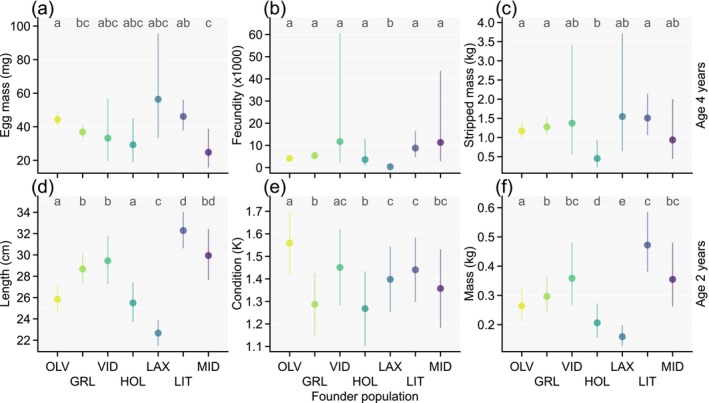
Multivariate animal model estimates for average genetic group effects with 95% confidence intervals (CI) for six traits and seven founder populations of the Icelandic Arctic charr breeding program. Estimates reflect breeding value differences among the founder populations (i.e., before selection) that we mean‐standardized for depiction to the average environmental effects expressed at the breeding station for the 2019 cohort, and for the traits expressed in mature females (a–c) to the maturation age of 4 years. In each panel, the same letter above each upper CI indicates groups with non‐significant pairwise differences at familywise *fdr* < 0.05. The population abbreviations in (b) refer to lakes (ending with *vatn*) and rivers (ending with *á* or *lækur*) with number of founders (N) in parentheses as OLV: Ölvesvatn (*N* = 126), LAX: Laxárvatn (*N* = 79), HOL: Hólalax (a previous aquaculture strain with uncertain Northern Icelandic origin; *N* = 23), GRL: Grenlækur (*N* = 138), VID: Víðidalsá (*N* = 11), MID: Miðfjarðará (*N* = 9), LIT: Litlaá (*N* = 45). Estimates for two additional founder populations holding minor genetic contributions (< 1%) from few founders (*N* ≤ 5) are not shown.

We had estimated and tested for differences in average additive genetic values among the founder populations and predicted their mean effects under standardized environmental conditions (Figure 5). For egg mass, only three population comparisons remained statistically significant when adjusted for the *fdr* < 0.05. OLV had a 20% (95% CI = 6%–31%) larger egg mass than GRL, both of which were the founder populations genetically dominating the selective breeding program (Figure 2a). The populations OLV and LIT had 80% (95% CI = 12%–105%) and 86% (95% CI = 14%–111%) larger egg mass, respectively, than MID (Figure 5a). Interestingly, OLV had a significantly smaller and LIT a significantly larger body length at age 2 years than MID (Figure 5d). In addition, OLV had a significantly larger body condition at age 2 years than MID (Figure 5e) and LIT a significantly larger body mass at age 2 years than MID (Figure 5e). Thus, OLV had a larger egg mass plus a larger body condition than MID, whereas LIT had a larger egg mass plus a larger body size than MID. No statistically significant differences in fecundity were detected among the three populations (Figure 5b).

For fecundity, only the LAX population yielded statistically significant comparisons. Specifically, fecundity was significantly smaller in LAX than in all other populations (only 3%–10% of others), which did not show significant pairwise differences among them (Figure 5b), and LAX was also the population with the largest egg mass estimate (albeit this estimate was very uncertain; Figure 5a) and the smallest length and mass at age 2 years (Figure 5d,f).

## 4 Discussion

We gained novel insights into the relationship between egg size and fecundity and how growth‐related traits expressed before and at sexual maturity, as well as age, associated with the two reproductive traits. We revealed complex genetic and environmental relationships between egg size and fecundity, and between each of these traits with growth traits. These findings contribute to our understanding of the egg‐size fecundity trade‐off, whether and how the two reproductive traits may evolve independently, co‐evolve with each other and with growth traits, and how environmental plasticity may alter the expression of all these traits. Because we detected the relationship in an admixed captive population while controlling for wild founder population effects (and also estimated the founder effects), the results probably generalize to several wild populations.

Our major results are that (i) genetic effects contribute more strongly to egg size than fecundity variation, (ii) the genetic correlation between egg size and fecundity is statistically indistinguishable from zero, (iii) the phenotypic and environmental correlations between egg size and fecundity are negative, relatively low, and significantly different from zero, (iv) growth traits expressed prior to and size at maturation show positive genetic correlations with both egg size and fecundity, and which may be the most striking result (v) growth traits expressed prior to and size at maturation show environmental (and overall phenotypic) correlations that are negative with egg size but positive with fecundity.

Together, these results in their combination suggest that a trade‐off between egg size and fecundity may exist at the environmental and phenotypic but not the genetic level, and this trade‐off appears as a dynamic parameter controlled by prior growth. Environmentally controlled increasing growth tends to associate with a decreasing egg size but increasing fecundity. We predict that the different sign of the genetic and environmental correlations with growth traits in combination with relatively independent genetic effects for egg size and fecundity enable expression of a wide range of phenotypic correlations (which are often used as tests for trade‐offs) between egg size and fecundity in both nature and captivity. A phenotypic life‐history trait correlation is often a dynamic parameter (e.g., D. A. Roff et al. 2002) and dynamics between egg size and fecundity depend, based on our results, on both the selection history across generations and the experienced environmental conditions within a given generation.

Our results on genetic and environmental covariances agree with previous suggestions in Atlantic salmon that the plastic response of both egg mass reduction and fecundity increase under beneficial growth conditions is the result of an environmental trade‐off (Bagenal 1969; Burton et al. 2020; Jonsson et al. 1996). However, other studies detected environmental effects on fecundity but not egg mass. For example, fecundity but not egg mass was affected by competition in vendace ( *Coregonus albula* ) (Wanke et al. 2017), and by feed ration in both zebrafish ( *Danio rerio* ) (Forbes et al. 2010) and Arctic charr (Frantzen et al. 2004). Quinn et al. (2004) found that egg mass was unaffected by growth rate, but that fecundity changed with growth rate at sea, although not with growth rate in freshwater for both Chinook and coho salmon. We estimated that fecundity variation was more consistently and strongly affected by growth traits than egg mass, which may explain some of the discrepancies among studies because it makes it statistically more difficult to detect growth effects on egg size than on fecundity.

### 4.1 Genetic Effects

Both egg size and fecundity have the potential to respond to selection due to their own genetic variances and their positive genetic covariances with growth traits. However, the non‐significant genetic correlation between egg size and fecundity—albeit negative but very close to zero—suggests an effective lack of strong direct evolutionary limitations between egg size and fecundity. Consequently, the two traits may evolve more independently than often assumed (like for many life‐history traits; Chang et al. 2024). This result contrasts with genetic correlation estimates in many species (Rollinson and Rowe 2015) and with expectations that hatchery practices induce co‐evolution towards smaller eggs via inadvertent selection for higher fecundity in salmon and charr (Heath, Heath, et al. 2003; Heath, Moya‐Laraño, and Fox 2003; Leblanc et al. 2016). Such co‐evolution would require either the presence of a negative genetic correlation between egg size and fecundity, or genetic correlations of opposite sign with additional evolving trait—none of which we detected here. Consequentially, to optimize maternal reproductive (clutch) fitness, natural or artificial selection may directly target egg size, fecundity, or both, whereby a non‐selected trait should not directly co‐respond with the other trait.

We estimated positive genetic correlations between growth traits and both egg size and fecundity, as well as a positive genetic correlation between body condition and fecundity. An expectation from this genetic correlation pattern is a unidirectional correlated response to selection of egg size and fecundity via selection on body size and to some extent of fecundity via selection on body condition (if selection occurs on body mass while ignoring length because it would be selecting for higher condition). This expectation was confirmed via the calculated genetic trends, although valid formal analyses of these trends are difficult (Hadfield et al. 2010; Postma 2006). These results still support the idea that body size co‐evolves with fecundity, as first suggested by Darwin (1871), and also with egg size. Due to this genetic correlation pattern, co‐evolution may also occur in the opposite direction. Selection on either egg size or fecundity is expected to induce co‐evolution of body size, and selection on fecundity also of body condition—both enable producing and storing larger and more eggs. Although the simultaneous co‐evolution via body size is expected to proceed in the same direction for egg size and fecundity, we expect it to change the relationship between egg size and fecundity because egg size correlates genetically to a lesser extent with body size than fecundity, and heritability differs between the two reproductive traits. Altogether, the complex genetic correlation pattern among growth traits and female reproductive traits suggests the presence of a possible concerted and beneficial co‐evolution of body size and shape with egg size and fecundity.

A quantitative genetic correlation between two traits indicates that some genetic variants underlying variation of both traits are either pleiotropic or in linkage disequilibrium. In this case, the genetic variants related to growth variation that are shared (or in linkage disequilibrium) with either egg mass or fecundity variation are likely to be different sets of genetic variants. This is because growth correlates genetically with egg mass and fecundity, while egg mass and fecundity do not correlate genetically with each other. This finding is intriguing, as growth variation may partially reflect resource acquisition variation, which is a trait typically assumed to correlate with many other traits that can be negatively correlated with each other via sharing a common resource (Van Noordwijk and De Jong 1986). We estimated that genetic effects for body size covary with those for both egg mass and fecundity, so that one may then assume that resource acquisition is mediating a negative genetic correlation between egg mass and fecundity. However, we also found egg mass and fecundity variation to be likely determined by independent genetic variation, as indicated by a lack of a genetic correlation between them, which is inconsistent with the assumption of resource acquisition as a common mediator trait. Thus, egg mass and fecundity may co‐evolve simultaneously in the same direction as growth rate (or body size at specific ages), but the shared genetic variants enabling this may differ between them.

The presence of a moderate positive genetic correlation between fecundity and body condition, but an effective lack of the correlation between egg mass and body condition one or two years prior to sexual maturity, is also interesting. Specifically, the body condition variation, as measured by Fulton's K, may reflect variation in energy reserves, body shape, or both. A positive genetic correlation between fecundity and body condition may thus firstly reflect the higher energy resources required to produce more eggs and (or) secondly the physical capacity to store more eggs at any given body length (assuming a larger body condition partly corresponds to a relatively larger body cavity). Both scenarios could occur simultaneously because visceral adipose tissue (an energy reserve) within the body cavity is recruited during gonadal development (Jørgensen et al. 2005; Manor et al. 2012) thereby freeing up space for eggs. If this concept holds true, it partially explains how potential space limitations for positive fecundity evolution (Einum et al. 2004) can be alleviated via concerted coevolution of fecundity and body condition (additional to the coevolution of body size discussed above). However, similar thoughts may apply to larger egg size because, everything else being equal, larger eggs also require more resources and space than smaller eggs. This, however, leaves the question of whether and how the required resources and space for larger eggs co‐evolve with egg size.

### 4.2 Environmental Effects

In contrast to the lack of support for a negative genetic correlation between egg mass and fecundity, we detected weak but significant negative phenotypic and environmental (residual) correlations between these two traits. This suggests that if egg size and number production rely on similar maternal resources, a resource allocation trade‐off between egg mass and fecundity exists for environmental effects. Through our multivariate analysis, we were able to put the negative environmental correlation between egg mass and fecundity into the context of an environmental correlation with growth prior to and size at maturation. In that regard, the environmental correlations between egg mass or fecundity with growth traits differed markedly between the two reproductive traits. Specifically, realized environmental plasticity for growth prior to maturation correlated negatively with that for egg mass and positively with that for fecundity. Furthermore, environmental plasticity for size at maturation did not correlate with environmental plasticity for egg mass but positively with environmental plasticity for fecundity. Interestingly, similar patterns have been observed in tree swallows ( *Tachycineta bicolor* ), which, unlike fishes, are determinate growers. In these birds, environmental quality induced plasticity in fecundity but not in egg mass (Pellerin et al. 2016). Under the premise that growth influences reproductive traits and not *vice versa*, one interpretation in fishes could be that environmentally governed growth plasticity exceeding the average genetically based growth potential (i.e., positive effects on growth plasticity) has effects that differ in their direction and temporal pattern between reproductive traits. Prior to maturation, positive growth effects act negatively on egg mass, and this effect ceases closer to maturation, but positive growth effects act positively on fecundity all the way until maturation. Thus, the recovered covariance pattern among growth, egg size, and fecundity matches expectations under the assumption that the experienced maternal environment acts as a predictor of the offspring environment quality (reviewed by Mousseau and Fox 1998).

Although the random environmental (residual) effects that cause growth plasticity in a captive environment (i.e., the breeding nucleus) may not mirror those in natural settings, their variation pattern can still provide relevant insights. In a breeding program, the expected variation of environmental effects is generally attributed to variation in environmental sensitivity to, and variation in, experienced unaccounted influences from various abiotic and biotic variables (Sae‐Lim et al. 2016). These variations may encompass those effects acting among equally aged individuals within natural settings. However, large temporal environmental differences in abiotic and biotic variables as existing in wild populations are usually intentionally minimized in captivity. Nevertheless, whereas effects may differ in magnitude, they may operate through similar mechanisms, such that growth reductions in either a captive or natural environment may incur similar responses of correlated life‐history traits.

### 4.3 Age vs. Growth Effects on the Relationship Between Body Size and Egg Mass or Fecundity

Based on the estimated fixed effects and covariance patterns, we can make predictions about how body size, related to either maturation age (fixed effect) or growth‐rate variation (covariance pattern) until each maturation age, affects egg mass or fecundity. When standardizing the fixed age effects to that for stripped body mass, we calculated predictive changes per 1% maturation‐age‐related (stripped) body mass increase of 0.39% egg mass increase and 1.45% fecundity increase. Utilizing predictive slopes from the estimated phenotypic covariance matrix (Appendix E; Table A6), we estimated per 1% growth‐related (stripped) body mass increase, 0.07% (± 0.01%) egg mass increase, and 0.53% (± 0.03%) fecundity increase. Thus, for the same proportional body mass increase, the increase in egg mass is 5.6 times and for fecundity 2.7 times larger when proceeding via maturation age than via growth‐rate variation. Lasne et al. (2018) also estimated a strong age‐related egg mass increase but a negative relationship between growth‐rate‐related body mass and egg mass in Arctic charr. This, altogether, suggests that maturation‐age‐related size differences have a several times larger importance on egg mass and fecundity variation than growth‐rate‐related size differences and lead to the expectation of a stronger association of maturation age than growth rate variation with egg mass and fecundity variation in Arctic charr.

Allometry differs when based on maturation‐age‐related or growth‐rate‐related body size. As a result of the predictions made above, when delaying maturation age from 3 to 4 years, we expect the ratio of egg mass to body mass (relative egg mass) to decrease (negative allometry) and the ratio of fecundity to body mass (relative fecundity) to increase (positive allometry). In contrast, we expect both ratios to decrease with body mass increase at each age (negative allometry). This different scaling of egg mass or fecundity with size, depending on the size variation source, emphasizes the importance of whether variation in (absolute or relative) egg mass or fecundity originates in differences of age, growth rate, or both. The implications of this may be considerable if our results hold more generally. Relevance may exist for several fields of research. These include making evolutionary inferences based on egg size or fecundity comparisons among populations or morphotypes with different growth rates (Power et al. 2005; Smalås et al. 2017; Takatsu et al. 2022) or for predicting effects of increasing water temperature on fecundity via decreasing body sizes (Barneche et al. 2018). Likewise, for fisheries management, assuming a false allometry can have severe consequences for predicting stock size (D. J. Marshall et al. 2021). Furthermore, whether maturation age, growth rate, or both are causing size variation and the type of allometry may matter for predicting short‐and long‐term effects of fishing on egg size and fecundity because fishing results in complex evolutionary and environmental effects on both maturation age and growth rate (Heino et al. 2015; Hutchings and Fraser 2008).

### 4.4 Comparisons With Previous Studies and Why This Is Difficult

Our interpretation that the maternal resource trade‐off between egg mass and fecundity exists at the environmental but not the genetic level aligns with many previous empirical findings (reviewed by Einum et al. 2004) and is consistent with the expectations of the theoretical model proposed by Smith and Fretwell (1974) but conflicts with empirical results summarized by Rollinson and Rowe (2015). Our phenotypic and genetic correlation estimates between egg mass and fecundity (*r*_*P*_ = −0.16, *r*_*G*_ = −0.04, respectively) are comparable to estimates in lizards and some fish species, but contrast with those in other fish species. For instance, a similar phenotypic correlation (*r*_*P*_ = −0.20) has been estimated in sand lizards (*Lacerta agilis*) (Ljungstrom et al. 2016). In rainbow trout ( *Oncorhynchus mykiss* ), both correlations have been estimated similarly to ours by Su et al. (1997) (*r*_P_ = −0.11; *r*_G_ = −0.10) and by D'Ambrosio et al. (2020) (*r*_P_ = −0.18, *r*_G_ = 0.01). In contrast, different results were obtained in coho salmon ( *Oncorhynchus kisutch* ) by Gall and Neira (2004), who estimated a lower negative phenotypic correlation (*r*_*P*_ = −0.27) and a much lower negative genetic correlation (*r*_*G*_ = −0.63) compared to our estimates. Also, a very low negative genetic correlation (*r*_*G*_ = −0.64) was estimated in Chinook salmon ( *Oncorhynchus tshawytscha* ) by Kinnison et al. (2001). Likewise, the environmental correlations between egg mass or fecundity and stripped body mass in rainbow trout were slightly weaker for egg mass (*r*_*R*_ = 0.21) than fecundity (*r*_*R*_ = 0.25) in the study of Su et al. (1997), and weaker for egg mass (*r*_*R*_ = 0.06) than fecundity (*r*_*R*_ = 0.18) in the study of D'Ambrosio et al. (2020) (calculated from Table 1), like what we observed in Arctic charr (egg mass, *r*_*R*_ = −0.02; fecundity, *r*_*R*_ = 0.30), whereas the same correlations in coho salmon (calculated from table 5 in Gall and Neira 2004) were stronger for egg mass (*r*_*R*_ = 0.14) than fecundity (*r*_*R*_ = −0.05).

Although between‐trait correlations are expected to vary among species and populations, correlation estimates can also vary depending on the methods used. Specifically, obtaining a negative correlation between two traits indicating a trade‐off may depend on whether covariation with one or several additional traits has been statistically adjusted (D. A. Roff 2002), for example by including fixed covariates (i.e., regression) or factors. In this study, we estimated genetic correlations while adjusting for maturation age and cohort effects. In contrast, some previous estimates of genetic correlations, including the negative estimate between egg mass and fecundity reported by Kinnison et al. (2001), were statistically adjusted for size variation by including body size as a covariate. For our data that show a strong selection response for body size (genetic trends in Figure 1d,f) it would be difficult to make valid comparisons if we had followed this approach. The reason is that the selection conducted for larger body size is expected to have also changed all genetically correlated traits. Whereas it is possible to statistically account for the selection trend when fitting body size as a response in an animal model (see methods), we are unaware of methods that can achieve this when including body size as a covariate. However, a failure to account for the selection trend in two traits that exhibit a genetic correlation may result in a selection‐distorted estimate.

Moreover, body size genetically covaries with both egg size and fecundity, and allometric scaling differs between egg size and fecundity (see above). Thus, how much variance will be removed from either egg size or fecundity, and how the covariance between the two changes when fitting body size as a covariate, will depend on the range of values covered by phenotypic body size. The presence of possible unaccounted different proportional age effects on all three traits (see above) further complicates the interpretation of a correlation estimate when fitting body size as a covariate; it may matter whether “size” refers to variation caused by age, growth rate, or both, and whether data are influenced by direct selection on maturation age, growth rate, or both. Similar challenges in data interpretation emerge for size‐standardized fecundity data (fecundity/body mass; aka *relative fecundity*) as is commonly used for studying reproductive effort.

An additional practical reason to be cautious when using body size as a covariate, instead of viewing it as a correlated trait, is that the response to selection depends on all relevant phenotypic and genetic (co)variances. Growth trait variation is highly relevant to natural and artificial selection for many possible reasons (reviewed by Arendt 1997). From an evolutionary or selective breeding perspective, it may thus be non‐optimal to estimate (co)variances (including a possible trade‐off) and selection responses on body‐size‐standardized reproductive‐trait values. Instead, it may be desirable to consider body size (or growth) as a relevant covarying trait, and this approach has recently been suggested to be favored (Chang et al. 2024). Employing a multivariate analysis, as we did here, we were able to disentangle and quantify (co)variances also for and with body size, thereby enabling a more complete understanding of the resource allocation trade‐off between egg mass and fecundity, of which the genetic and environmental covariances with body size are a critical component. However, genetic and environmental covariances may change with the environment (Sgrò and Hoffmann 2004; Stearns 1989) and the genetic covariances are prone to considerable change under varying allele frequencies of major loci (Agrawal et al. 2001). Thus, it would be interesting to assess multivariate trait performance across stronger environmental gradients and to determine the genetic architectures among traits.

### 4.5 Adjusting for Maturation Age

Building on ideas formulated above, we removed the covariance between maturation age and the traits expressed at maturation and thereby variation from many of the focal traits by fitting fixed effects for maturation age (and their interaction with cohort effects). We nonetheless chose this modeling approach because it enabled us to separate (co)variation caused by maturation age (i.e., a longer developmental duration) on traits expressed at maturity from (co)variation caused by growth rate on traits expressed at maturity. For example, everything else being equal, only variation in growth rate leads to variation in size at a common age, whereas a combination of growth rate and developmental duration variations leads to size variation across ages. Nonetheless, maturation age is a pivotal life‐history trait that trades off survival probability vs. reproductive success (Bernardo 1993; D. A. Roff 2002; Wells et al. 2017) and knowing (co)variance between relevant traits, including maturation age, is therefore highly relevant to understanding life history.

Treating maturation age as a fixed effect, as we did here, may therefore provide an incomplete insight into the phenotypic and genetic (co)variances relevant to the response to natural selection across maturation age classes. A more complete understanding may be obtained in future studies when direct growth rate data are available, instead of size‐at‐age data, as used here, and if these are modeled together with maturation age as an additional response trait. However, estimating growth rate requires more difficult‐to‐obtain longitudinal size records, which may ideally be split into phases from before and after maturation influences on growth rate (e.g., puberty growth spurt and fasting during spawning time). The reason is that both the environmental and the genetic correlation between size‐at‐age and maturation probability can reverse sign temporally in salmonids due to both effects of growth on maturation and effects of maturation on growth (Debes et al. 2021).

### 4.6 Mechanisms Connecting the Genotype and Phenotype

Although often emphasized to represent trade‐offs, negative genetic correlations may contribute negligible insight into possible mechanistic trade‐offs between traits (Charlesworth 1990) and studies on life‐history trade‐offs have been advised to encompass, among others, investigations of the physiology connecting phenotype and genotype (Stearns 1989). Our results suggest that a connection of phenotype and genotype may have to involve egg size, fecundity, and growth traits because of the lack of a genetic correlation between egg size and fecundity and possibly different sets of shared genetic variants between either egg size or fecundity and growth traits. In the case of Arctic charr, may such investigation, however, reveal genetic mechanisms associated with differences in allometry rather than direct trade‐offs.

Nonetheless, investigations of the physiology of the environmentally founded resource trade‐off between egg mass and fecundity may still contribute direct mechanistic insights. A focus of such investigations could be varying the quality of the environment related to growth rate and identifying the physiological processes decreasing egg mass but increasing fecundity under beneficial growth conditions (and the opposite under challenging growth conditions). Mechanisms for developmental adjustment in fishes have indeed already been suggested for fecundity, but, to the best of our knowledge, not for egg mass. Specifically, fecundity is assumed to be adjusted downwards via atresia of egg follicles (reviewed by Corriero et al. 2021; Saidapur 1978) and possibly upwards via recruiting additional oocytes (Tyler et al. 1996). In agreement with our results on a positive growth‐rate‐related and condition‐related environmental fecundity adjustment, atresia rate in fishes scales with female feed restriction severity (Kennedy et al. 2008; Scott 1962). Nonetheless, mechanisms of egg size variation among females appear underreported in fishes and may differ from mechanisms suggested, for example, in reptiles. In detail, in a *Sceloporus* lizard, gonadotropin (follicle‐stimulating hormone) injections decreased egg size and increased fecundity simultaneously, and a surgical fecundity decrease resulted in larger egg size (Sinervo 1990). However, attempts to link gonadotropins to egg size variation and to surgically reduce fecundity showed different results in rainbow trout. In rainbow trout, temporal expression patterns of gonadotropins could not be linked to smaller egg sizes under photoperiod‐induced shorter developmental duration of eggs (Bon et al. 1999). Furthermore, unilateral gonad removal did not dramatically lower fecundity because the remaining gonad compensated by recruiting additional oocytes, and egg size of the originally recruited oocytes was not increased (although the additionally recruited oocytes resulted in smaller eggs; Tyler et al. 1996). Thus, mechanisms of egg size adjustment remain less clear in fish than they are in reptiles and remain subject to research.

### 4.7 Genetic Group Effects

Although the traditional purpose of the mixed model with genetic group effects is a statistical control for genetically based differences among founder populations, or for individuals with unknown history (Quaas and Pollak 1981; Westell et al. 1988; Wolak and Reid 2017), the actual genetic group effect estimates may be interesting themselves. This is especially true for many fish species with a much more recent domestication history than many traditional domesticated species (15,000–1000 years; overview in MacHugh et al. 2017; Mignon‐Grasteau et al. 2005), as it is the case for Atlantic salmon (founded in the 1970s; Trygve Gjedrem 2010) or Arctic charr (founded in the 1980s; Eriksson et al. 2010; founded in the 1990s; this study); for many admixed domestication fish populations the often‐still‐existing wild founder populations are known (e.g., Trygve Gjedrem 2010; this study). The genetic group effect estimates for focal traits may thereby provide information firstly about potential target populations for establishing new or supplementing existing aquaculture breeding programs, and secondly potential insights into evolutionarily interesting patterns among populations. However, genetic population differences exist due to different allele frequencies that can result from either selection (evolutionarily highly interesting) or drift (evolutionarily less interesting) and differentiating statistically between the two causes is a major challenge.

We identified the LIT population as the population best meeting aquaculture breeding program objectives because it shows the highest genetic growth potential under large egg size and high fecundity. For evolutionary interesting relationships, we found statistical support that OLV has a genetic predisposition for larger egg size and body *condition* than MID, whereas LIT has a genetic predisposition for larger egg size and body *size* than MID. These genetic differences may indicate either genetic drift or different natural selection towards larger egg size in both OLV and LIT relative to MID. It is possible that, to physically account for the larger volume due to larger egg size, OLV evolved towards a larger body condition at small body size, whereas LIT evolved towards an overall larger body size. Assuming these differences emerged from natural selection, larger egg size should indicate challenging early offspring growth conditions in both OLV and LIT. OLV is an oligotrophic northern Icelandic lake population without access to temporary rich feeding at sea during summer months (two waterfalls block re‐entry from the sea), and achieving a large adult body size may be difficult, but changing body shape (condition) may not require excessive feeding. In contrast, LIT is a northern Icelandic anadromous river population with access to rich feeding at sea, and achieving a large body size is more easily possible. Thus, the estimated genetic population values for body condition and size meet theoretical expectations to accommodate a larger egg volume. However, a meaningful explanation for the lower fecundity in combination with the smallest length and mass of the northern Icelandic lake population LAX, relative to all others, may be more difficult to find. This lake is connected to the sea, so that a large body size could possibly be achieved. However, the inferred genetic predisposition for low fecundity and slow growth may indicate that the used LAX ancestors could be from a small non‐anadromous Arctic charr morph with low fecundity (e.g., Power et al. 2005; Smalås et al. 2017). The presence of different sympatric charr morphs, each occupying different ecological niches, complicates interpretations of how selection may have shaped life‐history traits.

## 5 Conclusions

Altogether, our results indicate that egg mass (egg size) and fecundity in Arctic charr each have a strong genetic basis that is, if at all, only weakly negatively genetically correlated. Environmental plasticity patterns for egg mass, fecundity, and growth match previously suggested maternal mechanisms of reducing egg mass and increasing fecundity under good environmental growth conditions. A resource allocation trade‐off between egg mass and fecundity thus appears to pertain to environmental effects. As a result, we expect that the sign of the observed phenotypic correlation between egg mass and fecundity of a population will be influenced primarily by environmentally governed growth plasticity. Future research targeted at either, or both, the genomic and physiological levels may contribute a more detailed insight into mechanisms of this growth‐mediated trade‐off.

## Conflicts of Interest

The authors declare no conflicts of interest.

## Supporting information


Appendix S1.

## Data Availability

Data for this study is available at the Dryad Digital Repository: https://doi.org/10.5061/dryad.3ffbg79x4.
