# Supplementary material for: Genetic and Environmental (Co)variation of Egg Size, Fecundity, and Growth Traits in Arctic Charr
Source: Evol Appl. 2025 Jul 18;18(7):e70135. doi: 10.1111/eva.70135 (PMC12271980; doi:10.1111/eva.70135)
Supplement: Supplementary file 1 — Appendix S1. [file EVA-18-e70135-s001.pdf]

Supporting Information for *Genetic and environmental (co)variation of egg size, fecundity, and growth traits in Arctic charr*

Appendix A. Outlier identification and data removal

To define "outliers" we fitted models as described in the main text, but including only three traits simultaneously. One model, we fitted to body length, condition, and mass at age 2 years, and another model, we fitted to reproductive traits of egg mass, fecundity, and stripped body mass. We defined "outlier" as the trait-specific model residual divided by the trait-specific residual standard deviation estimate having a *t*-statistic with *fdr* ≤ 0.05.

We excluded trait specific data for 22 individuals (0.02%) showing either length or mass outliers (but not both) and also excluded condition data for these individuals because condition is based on length and mass data. We excluded length, mass, and condition data for 44 individuals (0.03%) showing condition outliers, because this indicates mass, length, or both data record errors, or not noted abnormal shape or effects from not noted diseases. We also completely removed data from 18 individuals (0.01%) showing unusually small size ("stunted") for both length and mass, such as when growth was lastingly deprived by, e.g., diseases, deformities, or physiological malfunctions. We also excluded data from one individual showing an unusually small, stripped body mass (likely to be a data recording error), and from 22 individuals showing unusually low fecundity records (possibly due to incomplete egg stripping, which leads to negative fecundity bias).

Appendix B. Determining fecundity

We determined fecundity from information on I) female total "green" (unfertilised) egg wet mass, ii) female average green egg wet mass, and iii) female average eyed egg wet mass. We had average eyed egg wet mass for all female broodfish, but average green egg wet mass only for some females. However, relevant to the female is the green egg mass as this is the amount produced and carried by her. Fish eggs absorb water directly after fertilisation and increase both in volume and mass, so that eyed egg mass cannot be used directly to divide total green egg mass by average eyed egg mass. Instead, the average eyed egg mass needs to be converted first to average green egg mass. We therefore determined the amount of mass difference caused by water swelling between green eggs and eyed eggs. The visually assessed relationship between green and eyed eggs mass indicated a linear relationship. To estimate this relationship, we used a bivariate model with the responses of log of green egg mass (mg) and log of eyed egg mass (mg) recorded for 31 females of the 2019 cohort stripped in 2023. For each female, we obtained one record per female for average green egg mass based on 50 eggs, and 1-4 records per female for average eyed egg mass based on 25 eggs (totalling 65 average

records) because some females contributed eggs to several families, and we recorded eyed egg mass for each. To take advantage of the additional information due to the partly repeated design for female of average eyed egg mass, but not of average green egg mass, we fitted a model with an unstructured covariance matrix between traits for female identification and a constrained covariance matrix for the residuals. In this model, the female identification estimates average female egg masses (green and eyed) as random effects, each of their variances, and the covariance between them, whereas residual variance refers to measurement error across families for only eyed egg mass from females having several families. The residual covariance matrix was constrained to be zero for the between-trait residual covariance and fixed to a small value (0.0001 mg^2) for the residual variance of green egg mass (as this was non-repeated and could therefore not be estimated).

We then determined both trait means on the natural logarithmic scale for average eyed egg mass (± SE) to be 3.816 ± 0.010 and for average green egg mass to be 3.584 ± 0.013, i.e., egg swelling by water adsorption and development until the eyed egg stage resulted in an average effective mass increase of 26.1 %. This amount appears similar to estimates of mass increase by water swelling in Atlantic salmon (25%; Li et al., 1989). Repeatability of eyed egg mass measurements for the 31 females based on replicates among families (R_{eyed} ± SE) was R_{eyed} = 0.861 ± 0.028. The correlation estimate between eyed and green egg records ($r_{eyed,green}$ ± SE) based on the 31 females was $r_{eyed,green}$ = 0.898 ± 0.041. We estimated the regression coefficients for predicting green egg mass based on eyed egg mass via the estimated female identification (co)variances as slope β (± SE) = $cov_{green,eyed} / var_{eyed}$ = 1.0223 ± 0.1108, whose confidence interval included unity (95% CI = 0.8007-1.2439), and intercept α = -0.3165. When ignoring that the β estimate confidence interval included unity we may conservatively still assume that absolute egg mass affects the proportional mass difference change until the eyed egg stage (by, e.g., loss of egg mass by metabolism). Larger eggs relative to smaller eggs may then either adsorb less water during swelling or use more biomass for development until the eyed stage (i.e., have a lower gross conversion efficiency). Despite being non-significant, we conservatively took this estimate into account and predicted average green egg mass based on observed average eyed egg mass using the equation: average predicted $mass_{green}$ (mg) = $e^{(-0.3165 + \ln(\text{observed average mass}_{eyed}\ [mg]) * 1.0223)}$. We finally predicted fecundity (number of eggs) as total egg mass divided by predicted average green egg mass.

Appendix C. Estimated breeding value accuracy.

We calculated the estimated breeding value accuracy for each trait, which equals the expected correlation (r) between the estimated breeding value ($\hat{a}_i$) and the true breeding value (a_i), as:

$r_{(\hat{a}_i, a_i)} = \sqrt{1 - \frac{PEV(\hat{a}_i)}{\hat{\sigma}_a^2}}$, where $PEV(\hat{a}_i)$ is the prediction error variance, corresponding to the square of the standard error estimate of $\hat{a}_i$, and $\hat{\sigma}_a^2$ is the additive genetic variance estimate. All effect and parameter estimates used to calculate estimated breeding value accuracy were obtained from the multivariate animal model as reported in the main text.

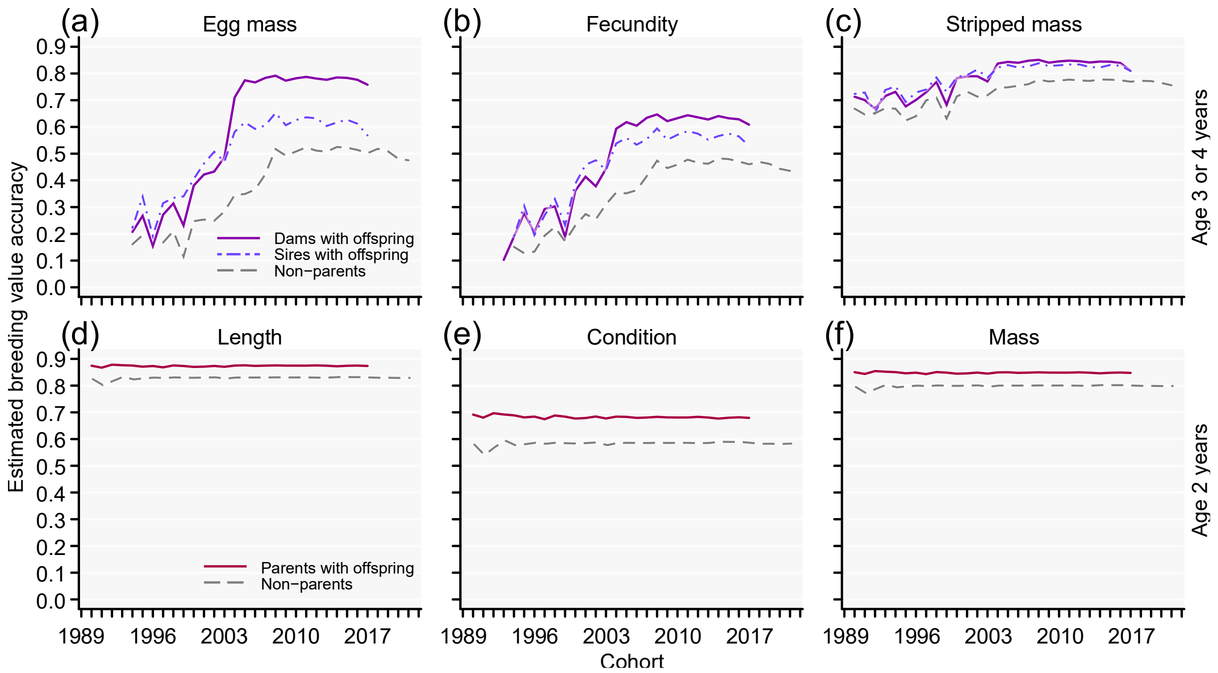

Figure A1. Trends for breeding value accuracy based on one common multivariate animal model for egg mass (**a**), fecundity (**b**), and body mass after egg stripping (**c**) at either 3 or 4 years, or for average body length (**d**), condition (**e**), and mass (**f**) of immature males and females at age 2 years. Accuracies are higher when the trait heritability is higher, genetic correlations with other informative traits are higher (in a multivariate model), and when more information about trait expression among relatives is available, so that estimates are higher for individuals with progeny and for individuals that have trait records. The latter is especially relevant for the female-limited traits (**a-c**), where trait expression in the breeding programme is limited to females used as dams and data records are only available for cohorts 2004-2019.

Appendix D. Estimated covariance matrices.

Table A1. Estimated phenotypic (co)variances (± standard error) among average egg mass, fecundity, and body mass after egg stripping of females that are first-time spawner at either 3 or 4 years, or for average body length, condition, and mass for males and females at age 2 years. Estimates are for scaled traits on the natural logarithm scale (except for condition).

	Egg mass	Fecundity	Stripped mass	Length	Condition	Mass
**Egg mass**	0.0092 ± 0.0072	0.0010 ± 0.0065	0.0104 ± 0.0050	0.0016 ± 0.0038	0.0059 ± 0.0029	0.0033 ± 0.0034
**Fecundity**	0.0010 ± 0.0065	0.0348 ± 0.0110	0.0106 ± 0.0061	0.0217 ± 0.0044	0.0052 ± 0.0033	0.0185 ± 0.0039
**Stripped mass**	0.0104 ± 0.0050	0.0106 ± 0.0061	0.0242 ± 0.0063	0.0201 ± 0.0032	0.0077 ± 0.0024	0.0183 ± 0.0029
**Length**	0.0016 ± 0.0038	0.0217 ± 0.0044	0.0201 ± 0.0032	0.0305 ± 0.0015	0.0072 ± 0.0010	0.0262 ± 0.0014
**Condition**	0.0059 ± 0.0029	0.0052 ± 0.0033	0.0077 ± 0.0024	0.0072 ± 0.0010	0.0227 ± 0.0012	0.0132 ± 0.0010
**Mass**	0.0033 ± 0.0034	0.0185 ± 0.0039	0.0183 ± 0.0029	0.0262 ± 0.0014	0.0132 ± 0.0010	0.0249 ± 0.0013

Table A2. Estimated common environmental (growing tank) (co)variances (± standard error) among average egg mass, fecundity, and body mass after egg stripping of females that are first-time spawner at either 3 or 4 years, or for average body length, condition, and mass for males and females at age 2 years. Estimates are for scaled traits on the natural logarithm scale (except for condition).

	Egg mass	Fecundity	Stripped mass	Length	Condition	Mass
**Egg mass**	0.0108 ± 0.0060	-0.0027 ± 0.0052	0.0050 ± 0.0044	NA	NA	NA
**Fecundity**	-0.0027 ± 0.0052	0.0157 ± 0.0086	0.0069 ± 0.0054	NA	NA	NA
**Stripped mass**	0.0050 ± 0.0044	0.0069 ± 0.0054	0.0114 ± 0.0058	NA	NA	NA
**Length**	NA	NA	NA	0.0592 ± 0.0161	0.0467 ± 0.0176	0.0618 ± 0.0174
**Condition**	NA	NA	NA	0.0467 ± 0.0176	0.1039 ± 0.0283	0.0713 ± 0.0216
**Mass**	NA	NA	NA	0.0618 ± 0.0174	0.0713 ± 0.0216	0.0723 ± 0.0197

Table A3. Estimated full-sib (co)variances (± standard error) among average egg mass, fecundity, and body mass after egg stripping of females that are first-time spawner at either 3 or 4 years, or for average body length, condition, and mass for males and females at age 2 years. Estimates are for scaled traits on the natural logarithm scale (except for condition).

	Egg mass	Fecundity	Stripped mass	Length	Condition	Mass
**Egg mass**	0.0092 ± 0.0072	0.0010 ± 0.0065	0.0104 ± 0.0050	0.0016 ± 0.0038	0.0059 ± 0.0029	0.0033 ± 0.0034
**Fecundity**	0.0010 ± 0.0065	0.0348 ± 0.0110	0.0106 ± 0.0061	0.0217 ± 0.0044	0.0052 ± 0.0033	0.0185 ± 0.0039
**Stripped mass**	0.0104 ± 0.0050	0.0106 ± 0.0061	0.0242 ± 0.0063	0.0201 ± 0.0032	0.0077 ± 0.0024	0.0183 ± 0.0029
**Length**	0.0016 ± 0.0038	0.0217 ± 0.0044	0.0201 ± 0.0032	0.0305 ± 0.0015	0.0072 ± 0.0010	0.0262 ± 0.0014
**Condition**	0.0059 ± 0.0029	0.0052 ± 0.0033	0.0077 ± 0.0024	0.0072 ± 0.0010	0.0227 ± 0.0012	0.0132 ± 0.0010
**Mass**	0.0033 ± 0.0034	0.0185 ± 0.0039	0.0183 ± 0.0029	0.0262 ± 0.0014	0.0132 ± 0.0010	0.0249 ± 0.0013

Table A4. Estimated additive genetic (co)variances (± standard error) among average egg mass, fecundity, and body mass after egg stripping of females that are first-time spawner at either 3 or 4 years, or for average body length, condition, and mass for males and females at age 2 years. Estimates are for scaled traits on the natural logarithm scale (except for condition).

	Egg mass	Fecundity	Stripped mass	Length	Condition	Mass
**Egg mass**	0.2461 ± 0.0278	-0.0157 ± 0.0206	0.0931 ± 0.0184	0.1043 ± 0.0151	0.0052 ± 0.0101	0.0839 ± 0.0138
**Fecundity**	-0.0157 ± 0.0206	0.1918 ± 0.0297	0.1295 ± 0.0196	0.1377 ± 0.0168	0.0598 ± 0.0112	0.1297 ± 0.0154
**Stripped mass**	0.0931 ± 0.0184	0.1295 ± 0.0196	0.3041 ± 0.0229	0.2942 ± 0.0134	0.1172 ± 0.0090	0.2751 ± 0.0124
**Length**	0.1043 ± 0.0151	0.1377 ± 0.0168	0.2942 ± 0.0134	0.3848 ± 0.0086	0.0945 ± 0.0056	0.3383 ± 0.0079
**Condition**	0.0052 ± 0.0101	0.0598 ± 0.0112	0.1172 ± 0.0090	0.0945 ± 0.0056	0.1880 ± 0.0061	0.1442 ± 0.0058
**Mass**	0.0839 ± 0.0138	0.1297 ± 0.0154	0.2751 ± 0.0124	0.3383 ± 0.0079	0.1442 ± 0.0058	0.3205 ± 0.0077

Table A5. Estimated random environmental (residual) (co)variances (± standard error) among average egg mass, fecundity, and body mass after egg stripping of females that are first-time spawner at either 3 or 4 years, or for average body length, condition, and mass for males and females at age 2 years. Estimates are for scaled traits on the natural logarithm scale (except for condition).

	Egg mass	Fecundity	Stripped mass	Length	Condition	Mass
**Egg mass**	0.1649 ± 0.0150	-0.0661 ± 0.0121	-0.0022 ± 0.0099	-0.0313 ± 0.0073	0.0143 ± 0.0062	-0.0186 ± 0.0069
**Fecundity**	-0.0661 ± 0.0121	0.3412 ± 0.0189	0.0852 ± 0.0116	0.0674 ± 0.0087	0.0277 ± 0.0073	0.0636 ± 0.0082
**Stripped mass**	-0.0022 ± 0.0099	0.0852 ± 0.0116	0.2080 ± 0.0127	0.0826 ± 0.0068	0.0636 ± 0.0055	0.0879 ± 0.0064
**Length**	-0.0313 ± 0.0073	0.0674 ± 0.0087	0.0826 ± 0.0068	0.1072 ± 0.0042	0.0367 ± 0.0028	0.0978 ± 0.0039
**Condition**	0.0143 ± 0.0062	0.0277 ± 0.0073	0.0636 ± 0.0055	0.0367 ± 0.0028	0.1547 ± 0.0031	0.0829 ± 0.0029
**Mass**	-0.0186 ± 0.0069	0.0636 ± 0.0082	0.0879 ± 0.0064	0.0978 ± 0.0039	0.0829 ± 0.0029	0.1070 ± 0.0038

Appendix E. Predictive slopes for egg mass and fecundity

A predictive slope (β) of trait *y* based on trait *x* can be estimated as $\beta = \frac{cov_{x,y}}{var_x}$. When the phenotypic (co)variances have been decomposed, the component (co)variance estimates can be summed to yield the phenotypic (co)variance estimates. Following this approach and estimating the standard errors using the delta method, we estimated predictive slopes for egg mass and fecundity based on body mass, length, and condition at age 2 years and based on stripped body size at maturation age of either 3 or 4 years. We back converted the slope and their error estimates by reversing the variance scaling and mean centring.

Table A6. Predictive slope estimates (β) for the responses (y) of egg mass or fecundity and based on the predictors (x) body length (mm), condition (K), or mass (g) at age 2 years, or stripped body mass (g) at either age 3 or 4 years. The slopes are on the natural logarithm scale of both the response and the predictor variables, except for the predictor variable of body condition, which is on the observed scale.

Level	Response	Predictor	β	se	z ratio
Phenotype	Egg mass	Length	0.1476	0.0267	5.53
Phenotype	Egg mass	Condition	0.0272	0.0100	2.71
Phenotype	Egg mass	Mass	0.0388	0.0072	5.41
Phenotype	Egg mass	Stripped mass	0.0744	0.0094	7.94
Phenotype	Fecundity	Length	1.4673	0.1034	14.20
Phenotype	Fecundity	Condition	0.3244	0.0408	7.95
Phenotype	Fecundity	Mass	0.3917	0.0293	13.37
Phenotype	Fecundity	Stripped mass	0.5315	0.0333	15.97
Full sib	Egg mass	Length	0.0597	0.1419	0.42
Full sib	Egg mass	Condition	0.1303	0.0633	2.06
Full sib	Egg mass	Mass	0.0396	0.0401	0.99
Full sib	Egg mass	Stripped mass	0.1654	0.0791	2.09
Full sib	Fecundity	Length	2.6819	0.5238	5.12
Full sib	Fecundity	Condition	0.3770	0.2389	1.58
Full sib	Fecundity	Mass	0.7210	0.1484	4.86
Full sib	Fecundity	Stripped mass	0.5490	0.2998	1.83
Common environment	Egg mass	Length	NA	NA	NA
Common environment	Egg mass	Condition	NA	NA	NA
Common environment	Egg mass	Mass	NA	NA	NA
Common environment	Egg mass	Stripped mass	0.1678	0.1315	1.28
Common environment	Fecundity	Length	NA	NA	NA
Common environment	Fecundity	Condition	NA	NA	NA
Common environment	Fecundity	Mass	NA	NA	NA
Common environment	Fecundity	Stripped mass	0.7611	0.4962	1.53
Additive genetic	Egg mass	Length	0.3119	0.0451	6.92
Additive genetic	Egg mass	Condition	0.0139	0.0271	0.51
Additive genetic	Egg mass	Mass	0.0777	0.0128	6.09
Additive genetic	Egg mass	Stripped mass	0.1173	0.0223	5.27
Additive genetic	Fecundity	Length	1.3465	0.1609	8.37
Additive genetic	Fecundity	Condition	0.5224	0.0963	5.42
Additive genetic	Fecundity	Mass	0.3926	0.0456	8.62
Additive genetic	Fecundity	Stripped mass	0.5337	0.0760	7.03
Random environment	Egg mass	Length	-0.3354	0.0796	-4.21
Random environment	Egg mass	Condition	0.0466	0.0202	2.30
Random environment	Egg mass	Mass	-0.0515	0.0192	-2.69
Random environment	Egg mass	Stripped mass	-0.0040	0.0184	-0.22
Random environment	Fecundity	Length	2.3648	0.2970	7.96
Random environment	Fecundity	Condition	0.2939	0.0768	3.83
Random environment	Fecundity	Mass	0.5768	0.0721	8.00
Random environment	Fecundity	Stripped mass	0.5136	0.0652	7.87
